# The Laws of Natural Deduction in Inference by DNA Computer

**DOI:** 10.1155/2014/834237

**Published:** 2014-07-03

**Authors:** Łukasz Rogowski, Petr Sosík

**Affiliations:** ^1^Research Institute of the IT4Innovations Centre of Excellence, Faculty of Philosophy and Science, Silesian University in Opava, 74601 Opava, Czech Republic; ^2^Department of Math and Computer Science, University of Lodz, 90238 Łódź, Poland

## Abstract

We present a DNA-based implementation of reaction system with molecules encoding elements of the propositional logic, that is, propositions and formulas. The protocol can perform inference steps using, for example, *modus ponens* and *modus tollens* rules and de Morgan's laws. The set of the implemented operations allows for inference of formulas using the laws of natural deduction. The system can also detect whether a certain proposition *a* can be deduced from the basic facts and given rules. The whole protocol is fully autonomous; that is, after introducing the initial set of molecules, no human assistance is needed. Only one restriction enzyme is used throughout the inference process. Unlike some other similar implementations, our improved design allows representing simultaneously a fact *a* and its negation ~*a*, including special reactions to detect the inconsistency, that is, a simultaneous occurrence of a fact and its negation. An analysis of correctness, completeness, and complexity is included.

## 1. Introduction

Deoxyribonucleic acid (DNA) computing is the computational paradigm which uses organic molecules instead of traditional computer technologies to store and manipulate data. It is an interdisciplinary crossroad of biotechnology, nanotechnology, and computer science, based on manipulations with DNA strands in special laboratory conditions. The biggest advantage is the massive parallelism of reactions which led us to making trillions of similar calculations at the same moment [1, 2].

The idea was first implemented in 1994 by L.M. Adleman, a computer scientist from the University of Southern California. He presented a concept of how to solve in that way a well-known NP-complete problem HPP—how to find a Hamiltonian path in a graph. Nodes and edges of the graph were encoded by special single-stranded DNA molecules and then mixed in a test tube. All possible paths were created during the reaction and then only the Hamiltonian paths were filtered out by standard laboratory steps. The experiment was tested in laboratory for a 7-node graph [3]. This idea of solving problems (creating every possible candidate solution and then checking in parallel if they meet all required conditions) is called* computing by carving* and recently it is utilized not only in DNA computing [4].

After Adleman's work, many following ideas of solving computational problems by DNA were presented. There were ideas about how to solve another NP-complete problem called SAT [5–7], to simulate finite automata [8, 9], pushdown automata [10], data compression algorithms [11], logical gates [12, 13], and more. DNA computing can be successfully combined with other bioinspired computing techniques as the evolutionary computing, quantum computing, particle swam optimization, and others [14]. The research which we are going to present here was primarily inspired by Benenson's idea of implementing simple 2-state and 2-symbol finite automata using the concept of* splicing* (alternately connecting complementary parts of DNA molecules after cutting them by a specific restriction enzyme) [8, 9]. It was extended in subsequent papers for more states and more symbols [15, 16], especially, thanks to a new idea of splicing with the possibility of utilizing two or more restriction enzymes in the same mixture [17]. As an important inspiration we took also the ideas of implementing simple logical inference by splicing systems, suggested theoretically [18] and with positive laboratory tests [19]. Both of them presented the concept of deduction from one-valued facts (all formulas encoded by DNA molecules are assumed to have the* true* value) including conjunction, disjunction, and simple conditionals. There is a possibility to ask the system whether a certain fact can be deduced or not. A suggestion of how to add negation (*false* value of facts) was shown in [18] but in that case it was impossible to use conditional reactions. In this paper we propose a novel implementation using both fact values (*true* and* false*). Some preliminary concepts were already suggested by the first author in [20].

The main advantage of our protocol is the possibility to implement logic axioms and laws of natural deduction, which was impossible in previous implementations [18, 19]. They include (i) completion of the* modus ponens* rule with the* modus tollens*: even a simple implication *a* → *b*, implemented by a single molecule, is reversible: given *a*, our system deduces *b*, and, given ~*b*, the system deduces ~*a*; (ii) implementation of de Morgan's laws allowing to create a valid and compact system compatible with the laws of classical logic; and (iii) implementation of proofs by contradiction which are natural in many applications of logic.

## 2. Basic Concepts and Terms

### 2.1. Mathematical Background

Elementary logic variables are simply called* facts* or* terms*. Each of them can have two values:* true* or* false*. Every formula consists of facts and logical connectives: conjunction ∧ (,,and”), disjunction ∨ (,,or”), negation ~ (,,not”), and conditional → (,,if…, then…”), and parentheses is called logical sentence. Using both values of facts, it is possible to conclude (by conditionals and the* modus ponens* rule) new facts which can also adopt both truth values. In the system which is going to be presented the exact implementation of parentheses and disjunction is impossible, so every formula has to be written in a special form based on conjunction and implication.

The conjunctive normal form (CNF) is a way of simplifying logical formulae to be a conjunction of clauses, where a clause is a disjunction of literals. For example the following formulae are in CNF:~*a*∧(*b*∨*c*), (*a*∨*b*)∧(~*b*∨~*c*∨*d*)∧~*d* whilst the next ones are not in CNF:~(*a*∨*b*), (*a*∧*b*)∨*c*. To convert every logical formula to CNF, we need the following laws of classical logic:

-  ~(~**a**) ≡ **a** (rule of double negation),
- (~(**a**∧**b**))≡((~**a**)∨~**b**) (the first de Morgan's law—negation of conjunction),
- (~(**a**∨**b**))≡((~**a**)∧~**b**)(the second de Morgan's law—negation of disjunction),
- (**a**∧(**b**∨**c**))≡((**a**∧**b**)∨(**a**∧**c**)) (distributive conjunction by disjunction),
- (**a**∨(**b**∧**c**))≡((**a**∨**b**)∧(**a**∨**c**)) (distributive disjunction by conjunction).

To convert every formula written in CNF to our special form, where we want to replace disjunctions by implications, we need some more laws:

- (**a** → **b**) ≡ ((~**b**) → ~**a**) (rule of contraposition,* modus tollens*),
- (**a**∨**b**)≡((~**a**) → **b**) (disjunction represented by negation and conditional).

When the system has every formula rewritten in that way, it can autonomously process it and deduce new facts and formulas using* modus ponens*,* modus tollens,* and de Morgan's laws. It is also possible to ask the system whether a given value of a certain fact was deducted or not.

### 2.2. Elementary Operations with DNA

DNA (deoxyribonucleic acid) is a long polymer made from repeating units. Those units are called nucleotides. They are composed of sugars (deoxyribose), phosphate groups, and nucleobase attached to the sugars. They differ from each other only by the last part and there are four possibilities: adenine, cytosine, guanine, and thymine, abbreviated using the letters A, C, G, T. Most DNA molecules are double-stranded helices consisting of two long polymers. These strands bind in opposite directions to each other. One end has 5′-OH group whilst the second one has 3′-OH group. The bonds are subject to the Watson-Crick complementarity rule: adenine is always connecting with thymine by double hydrogen bonding and cytosine with guanine by triple hydrogen bonding. Basic laboratory operations on DNA, which are mostly utilized for DNA computing, are as follows.

**Figure 1:**
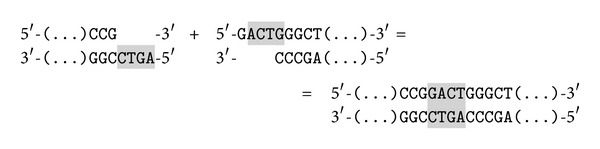
Annealing and ligation.

*(i) Annealing and Ligation.* When parts of two DNA sequences are complementary to each other, the molecules can bind and create a double-stranded molecule, see Figure 1. It is called* annealing*. A specific enzyme utilized to catalyse annealing between* sticky ends* (short single-stranded ends of double-stranded molecules) is named ligase.

*(ii) Cutting*. Some enzymes in specific laboratory conditions (i.e., temperature) recognize a certain short sequence of nucleotides, attach to this sequence, and cut the molecule in exact place in or after that sequence (operation opposite to ligation). Some enzymes leave molecules with sticky ends. For example enzyme Bse*XI* (which we are using in our system) recognises sequence 
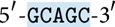
 and cuts the molecule after 8 and 12 nucleotides, respectively. The recognised sentence is marked by a color background in the following figure,  N denotes any possible value of a nucleotide and | marks the cutting point.


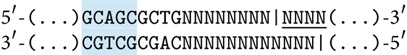


*(iii) Gel Electrophoresis*. A laboratory procedure for separating DNA molecules by their length. It uses an electrical field and gel matrix. The negatively charged molecules move towards a positive electrode. The gel matrix allows shorter DNA fragments to migrate more quickly than larger ones. It is possible to distinguish molecules by their length, including even the one-nucleotide length differences. Some modern versions as the* capillary electrophoresis* are even more sensitive and efficient.

## 3. A Relation to Splicing Systems

The DNA implementation of logic operations described in this paper depends heavily on the iterated operations of DNA ligation and cutting by a restriction enzyme and the combination of both. These operations have been previously used in models of DNA computing as sticker systems, ins-del systems, splicing systems, and many more; see, for example, [1, 2] for more details. Splicing systems represent the most characteristic use of the combination of both operations. In this section we briefly describe their principles. Assume two restriction enzymes (e.g.,* Taq*I,* Sci*NI) with the following cutting sites:


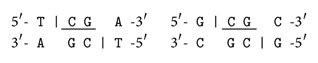


**Figure 2:**
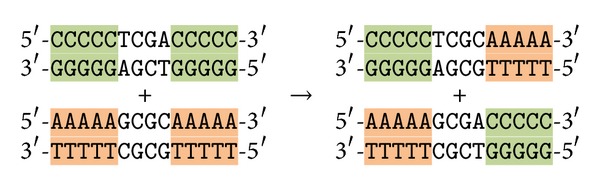
The operation of splicing.

Notice that both sites have the common pair of nucleotides CG in the middle. Let us furthermore consider two DNA molecules containing these sites. They can both be cut by their respective enzymes, and the four resulting molecules with sticky ends can then crossover anneal, producing two new molecules. Graphically, the operation of* splicing* is depicted in Figure 2.

Formally, this operation is defined using the formal language framework. Let *V* be a generalized alphabet of symbols forming DNA strands, that is, not necessarily the {*A*, *C*, *G*, *T*} alphabet but an arbitrary one. The operation of* splicing* is defined as

(1) (x,y)⟹  (z,w) iff    x=x1u1u2x2y=y1u3u4y2z=x1u1u4y2w=y1u3u2x2,

where (*u*_1_, *u*_2_; *u*_3_, *u*_4_) is a splicing rule and *x*_1_, *x*_2_, *y*_1_, *y*_2_∈V* (where V* is a set of all strings over the alphabet *V*). Furthermore, *u*_1_ and *u*_3_ share a common suffix, say *v*, so that the cross-annealing of *u*_1_ with *u*_4_ and *u*_2_ with *u*_3_ as in the previous figure could happen. This operation is interpreted in such a way that molecules *x* and *y* produce molecules *w* and *z*.

It is known that the operation of splicing is powerful and that the splicing systems with sets of splicing rules can generate all recursively enumerable languages under various restrictions. However, the use of splicing to implement logic operations is not mentioned in the relevant literature as [1, 2]. In this paper we use almost identical combination of operations, although we do not require that a combination of cutting by enzyme and subsequent ligation must be done at each computational step. We independently allow some cutting steps and some annealing steps.

## 4. A Novel DNA Implementation of Logic Operations

The implementation which we are going to present is based on a splicing system which was already explained. The ligase is utilized to catalyse the annealing of complementary parts of molecules, on one hand. On the other hand, we use the restriction enzyme named Bse*XI* which leaves 4-nucleotides sticky ends; see Section 4.2.

The presented system is fully autonomous which means that a human assistance is needed only to prepare constituents of reaction, to mix them in a test tube, and to read an answer by the electrophoresis after all the reactions have taken part. There is no need to add or remove any substances during the reaction. The restriction enzyme has to be added just once and it autonomously finds molecules which have to be cut.

### 4.1. Basic Encoding

Logic variables and their values are encoded by unique sequences of 4 nucleotides. Single-stranded sequences assigned to the same variable with different values (*true* and* false*) are complementary to each other (e.g.,  **a** is always complementary to ~**a**). The short DNA sequence recognised by the restriction enzyme (3′-GTCG-5′) and some special sequences which are complementary to themselves (e.g., 3′-AATT-5′) have to be excluded from that class. Finally it is possible to use exactly 119 unique variables encoded by different 4 tuples.

To make examples easier to understand, sequences will be presented in the following way:


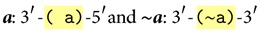


These unique 4-nucleotide sequences will be used not only for terms but also as a part of conditional rules or questions asked to the system.

#### 4.1.1. Representation of Terms

Molecules representing terms share a common starting sequence and a constant length. They also contain the part recognised by Bse*XI*. They differ from each other only by sticky ends which were already mentioned. For example molecules representations of  **a** and ~**a** look like


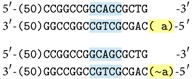


#### 4.1.2. Terminal Molecule

There is one special molecule which is utilized in every reaction; it is called the* terminal molecule*. It has the same beginning as the molecules representing terms but it ends earlier, in the middle of the sequence recognised by the restriction enzyme:


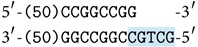


### 4.2. The Reaction of Inconsistency

**Figure 3:**
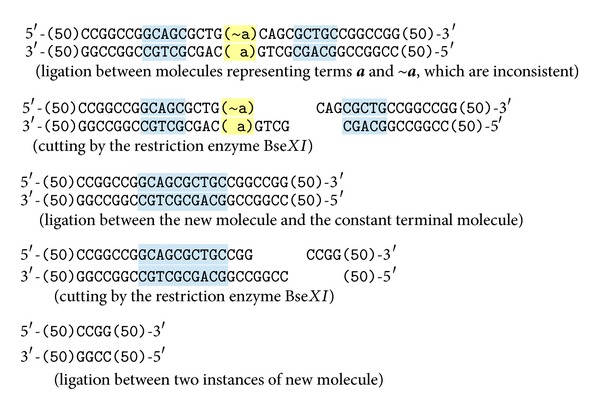
The reaction of inconsistency.

All the molecules encoding formulas in a test tube are assumed to exist in conjunction. Therefore, the existence of both values of the same variable at the same reaction (e.g.,  **a** and ~**a**) means a logical inconsistency. If it happens, the following reaction will start due to the complementarity of **a** and ~**a**, and the system will detect and signalize this situation by the existence of a special molecule. The reaction steps are shown in Figure 3.

As a result we get a molecule with the length of 104 nucleotides in each strand. After terminating reactions, it is possible to read the length of every molecule by the electrophoresis. If a molecule with this length occurs in the test tube, it means that an inconsistency occurred during the reactions. In that case the set of encoded formulae is unsatisfiable, and any formula can be potentially derived from it by the deduction laws.

**Figure 4:**
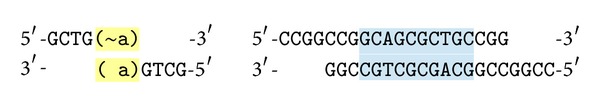
Possible artifact molecules.

Observe that the enzyme Bse*XI* can act also differently as the binding sites in some of the molecules described above are duplicate and symmetric. Therefore, the following artifact molecules can be produced, see Figure 4.

The first of these molecules has the same sticky ends as the terminal molecules and, therefore, it can compete with them in the above reactions. However, since we assume an abundant amount of each species of molecules is present during the reactions; this will not prevent the correct reactions to take place. The second artifact molecule has self-complement sticky ends and hence it can eventually iterate itself. However, it cannot interfere with other programmed reactions.

### 4.3. A Simple Conditional

The simplest inference step means just one fact in antecedent and just one fact in consequent of an implication. The contraposition rule implies that in one conditional there are two possibilities of inference; for example,    **a** → **b** means also ~**b** → ~**a**. The molecule representing this rule looks like


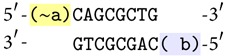


The first sticky end is always complementary to the antecedent and the second one is identical to the consequent. It is important to know that if we rotate view of this molecule we obtain the molecule representing contraposition conditional (without changing the orientation of DNA strands):


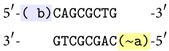


#### 4.3.1. Reaction of Inference for *a* and *a* → *b*

**Figure 5:**
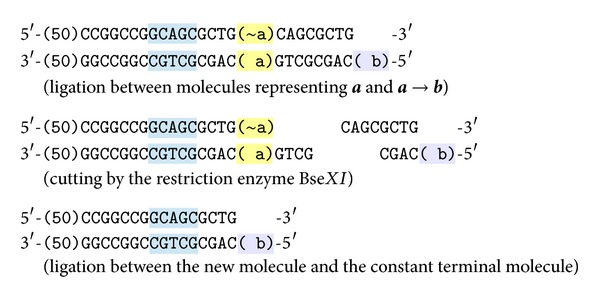
The reaction of inference for** a** and** a **→** b**.

If we mix in one test tube molecules representing* true* value of fact  **a**, the conditional  **a** → **b**, the terminal molecule, ligase, and the restriction enzyme Bse*XI*, the reaction steps are shown in Figure 5.

As a result we get the molecule representing the fact  **b**. This molecule can take part in subsequent reaction steps with other molecules. If initially, instead of   **a**, the molecule representing ~**b** was in test tube, analogous reactions would create the molecule representing ~**a**.

### 4.4. Conjunction in Inference

Inference rules containing conjunction can be implemented as a conditional with conjunction in any part of the conditional: antecedent and consequent.

- The conjunction in consequent has to be divided into two simple conditionals. As it was mentioned in previous explanation of mathematical laws, for example, (**a** → (**b**∧**c**)) ≡ ((**a** → **b**)∧(**a** → **c**)). Using the first de Morgan's law and the rule of contraposition we get ((~**b**∨~**c**)→~**a**) that also can be divided to two simple conditionals (~**b** → ~**a**) and (~**c** → ~ **a**). The molecular representation of this pair of conditionals and the pair mentioned in the previous sentence is the same because of* modus tollens*. We need two molecules to represent conjunction between two elements, and three molecules to represent conjunction between three elements and so on for more elements.
- The conjunction in antecedent requires a construction of a new molecule which extends the one called simple conditional. It implements the inference using the first de Morgan's law and the rule of contraposition. For the conditional (**a**∧**b**) → **c** (which also means ~**c** → (~**a**∨~**b**)) the molecular implementation is

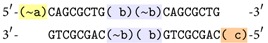


When a more complex conjunction has to be considered, that is, (**a**∧**b**∧**c**) → **d**, it can be decomposed to simpler ones by adding a new fact  **e** and setting (**a**∧ **b**) → **e**, (**e**∧ **c**) → **d**.

#### 4.4.1. Reaction of Inference for *a*, *b* and *a*∧*b* → *c*

**Figure 6:**
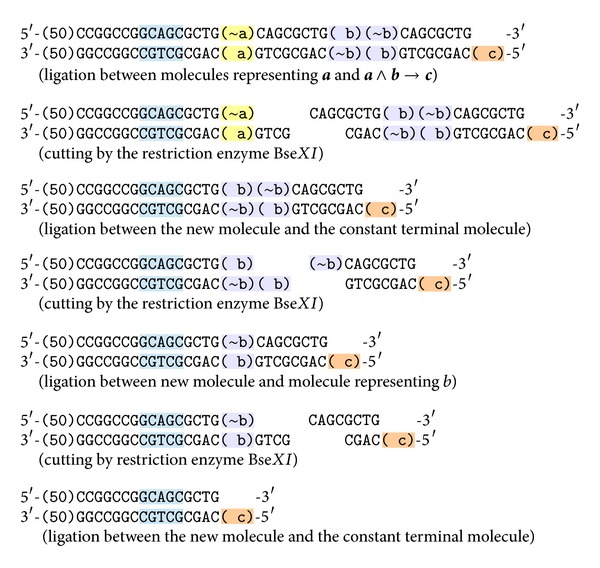
The reaction of inference for** a**,** b** and** a **∧** b **→** c**.

If we mix in one test tube molecules representing* true* value of fact  **a**,* true* value of fact  **b**, the conditional (**a**∧**b**) → **c**, the terminal molecule, ligase, and the restriction enzyme Bse*XI*, the reaction steps are shown in Figure 6.

As a result we get the molecule representing the fact  **c**. This molecule can take part in subsequent reactions with other molecules.

#### 4.4.2. Reaction of Inference for ~*c* and *a*∧*b* → *c*

**Figure 7:**
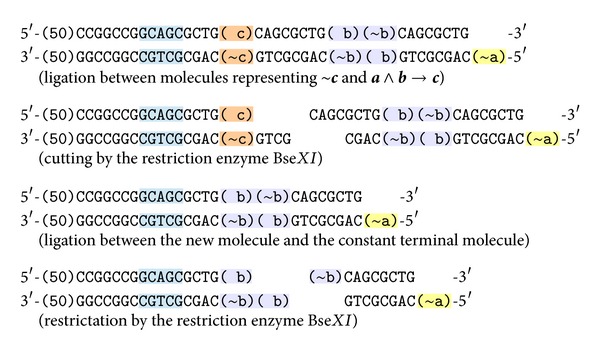
The reaction of inference for ~**c** and a ∧** b **→** c**.

Assume that, instead of  **a** and  **b**, the molecule representing ~**c** was initially in test tube. If we mix in one test tube the molecules representing* false* value of fact  **c**, conditional (**a**∧**b**) → **c**, the terminal molecule, ligase, and the restriction enzyme Bse*XI*, the reaction steps are shown in Figure 7.

As a result we get the molecule representing the conditional **b** → (~**a**), which means exactly  **a**∧**b** due to the law representing conjunction by the negation and conditional. This molecule can take part in subsequent reactions with other molecules; particularly if there is a molecule representing fact  **b**, it will deduce ~**a**, and, in the opposite way, if there is a molecule representing fact  **a** it will deduce ~**b**.

### 4.5. Disjunction in Inference

The disjunction also can be implemented in both parts of conditional: antecedent and consequent.

- The disjunction in antecedent has to be divided into two simple conditionals, in the similar way like conjunction in consequent; for example ((**a**∨**b**) → **c**)≡((**a** → **c**)∧(**b** → **c**)). Using the second de Morgan's law and the rule of contraposition we get (~**c** → (~**a**∧~**b**)) which can also be divided into two simple conditionals (~**c** → ~**a**) and (~**c** → ~**b**). The molecular representation of this pair of conditionals and the pair mentioned in the previous sentence is the same because of* modus tollens*. We need two molecules to represent disjunction between two elements and three molecules to represent disjunction between three elements and so on for more elements.
- The consequent part needs a construction of a new molecule, similar to the one present in conjunction description. It saves proper inference using second de Morgan's law and the rule of contraposition. For conditional  **a** → (**b**∨**c**) (which also means (~**b**∧~**c**)→~**a**) it is:

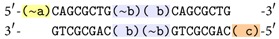


Examples of the corresponding reactions are similar to those presented in the section concerning conjunction. If we mix in one test tube the molecules representing* true* value of the fact  **a**, the conditional  **a** → (**b**∨**c**), the terminal molecule, ligase, and the restriction enzyme Bse*XI*, we will eventually receive the molecule representing ~**b** → **c** which, according to the laws of classical logic, represents  **b** *∨* **c**. If we mix in one test tube the molecules representing* false* value of the fact  **b**,* false* value of the fact  **c**, the conditional  **a** → (**b**∨**c**), the terminal molecule, ligase, and the restriction enzyme Bse*XI*, we will receive the molecule representing * false* value of fact  **a**. Every new molecule can take part in subsequent reactions.

### 4.6. Asking Questions

Our reaction system can be asked questions like “is it possible to deduce a certain value of a given fact starting from the conditions which we know?" If we ask a question whether **a** is true and we would not receive positive answer, it does not mean that the system knows something about ~**a**. It just means that  **a** cannot be deduced. It is possible to ask more than one question at the same moment but the molecules representing the questions must differ from each other by their lengths (as answers are distinguished by lengths). It is impossible to ask two questions for different truth values of the same variable (e.g.,  **a**? and ~**a**?) because the system will treat it like disjunction and, according to the law of the excluded middle, the answer will be always positive.

Every question contains a sticky end identifying variable (the complementary part) and a unique 4-nucleotide sequence identifying question (which has to belong to the class of sequences complementary with themselves, excluding 3′-GGCC-5′ which has been already used for the reaction of inconsistency). The question is then completed with an arbitrary sequence (not containing the binding site of the restriction enzyme) to get a unique length of the molecule. For example, the molecules representing  **a**? and ~**b**? can look like:


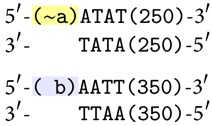


#### 4.6.1. Reaction for *a* and the Question *a*?

**Figure 8:**
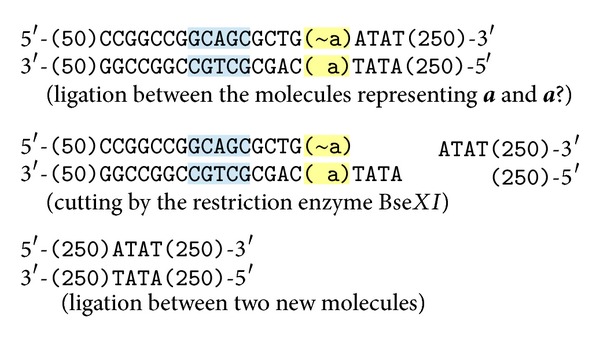
The reaction for a and the question** a**?

If we mix in one test tube molecules representing* true* value of the fact  **a**, the question  **a**?, ligase and the restriction enzyme Bse*XI*, the reaction steps are shown in Figure 8.

As a result we get a molecule with the length of 504 base pairs (bp). This length means a positive answer for the question  **a**?. Absence of this molecule would mean that nothing is known about   **a**. Analogously, the existence of a molecule of 704 bp long would mean a positive answer to the question  **b**?. As it was mentioned, this kind of questions does not exclude each other.

## 5. Soundness and Completeness

In this subsection we show that (a) if a positive answer to a question is deducible from the initial set of formulas, then there are reactions which would eventually produce the corresponding molecule of length 504 bp, and (b) if the system gives a positive answer, then it is truly deducible from the initial set of formulas. We can take for granted three simple reactions (based on splicing and possible only when some molecules have complementary sticky ends): the reaction of inconsistency (presented in Section 4.2), the simple inference (Section 4.3.1), and the positive answer reaction (Section 4.6.1). Their correctness was already checked in similar laboratory experiments [17, 19].

In the mathematical way, let** B** be the set of all known basic terms (atomic facts with their values) present in a test tube, let** T** be the set of other formulae in the test tube (implications and the rest which can be rewritten by implications using the laws of classical logic that is, (**a**∨**b**)≡(~**a** → **b**)), and let** Q** be the set of questions in the test tube. Accordingly let |**B**|, |**T**|, and |**Q**| mean the quantity of their elements.

We define the formula of inconsistency as** INCONS** *≡* (*∃*(**a** ∈ **B**)∧(~**a** ∈ **B**)). An inference of this formula implies that it makes no sense to analyze further answers and that the ones already received may be incorrect. Now the evidence is focused on situation when the inconsistency does not occur and only this situation can be considered as finished with proper deducible answer.

For the proof we treat sets** B** and** Q** as arbitrary, but assume that** B** is consistent; that is, the formula** INCONS** is false for** B **(in the opposite case the system immediately runs the reactions in Section 4.2). The proof of completeness is based on mathematical induction on the number of elements in** T**.

1. Let |**T**| = 0; then two cases are possible and the system reacts correctly in both of them by Sections 4.3.1 and 4.6.1:
  - (*∃* (**a** ***∈* B**) *∃* (**b** ***∈* Q**) (**a** = **b**)) ⇒ reactions give the positive answer molecule;
  - ~(*∃* (**a** ***∈* B**) *∃* (**b** ***∈* Q**) (**a** = **b**)) ⇒ reactions do not give the positive answer molecule.
2. Let us assume that the system gives the correct answer for a given set** T**. We show that, when any new implication **i** is added, the system of reactions described in Section 4 will reduce the situation to the correctness for** T**. Let the new set be marked as** NT**, where** NT** =** T** *∪*  {**i**} and |**N** **T** | = | **T** | +1. We analyse all the possibilities of construction implication **i** and its resolution by the reaction in Section 4:
  - **i** ≡ (**a** → **b**):
    - (**a** ∈ **B**)∧(~**b** ∉ **B**)⇒(**B**′ = **B** ∪ {**b**})∧(**N** **T**′ = **N** **T**–{**i**} = **T** ∪ {**i**}–{**i**} = **T**),
    - (**a** ∉ **B**)∧(~**b** ∈ **B**)⇒(**B**′ = **B** ∪ {~**a**})∧(**N** **T**′ = **N** **T**–{**i**} = **T** ∪ {**i**}–{**i**} = **T**),
    - (**a** ∈ **B**)∧(~**b** ∈ **B**)⇒** INCONS**,
    - (**a** ∉ **B**)∧(~**b** ∉ **B**)⇒ there is no reaction in this situation so there is no impact for the final answer; we can also treat this case as (**NT**′=**NT**–{**i**}=** T** *∪*   {**i**}–{**i**} =**T**). If **a** or ~**b** appears later as a result of new formulae added to** T**, then **i** can be readded and treated as in paragraphs (i), (ii), or (iii) above.
  - **i** ≡ (**a** → (**b**∨**c**))≡(**a** → (**c**∨**b**)):
    - (**a** ∈ **B**)⇒(**i**′ = ~**b** → **c**) ⇒ (then further analysis is like that in paragraph (a)),
    - (~**b** ∈ **B**)⇒(**i**′ = **a** → **c**)⇒ (then further analysis is like that in paragraph (a)),
    - (~**c** ∈ **B**)⇒(**i**′ = **a** → **b**)⇒ (then further analysis is like that in paragraph (a)).
    - (**a** ∈ **B**)∧(~**b** ∈ **B**)∧(~**c** ∈ **B**)⇒**INCONS**,
    - (**a** ∉ **B**)∧(~**b** ∉ **B**)∧(~**c** ∉ **B**)⇒ there is no reaction in this situation so there is no impact for the final answer, we can treat that as (**NT**′=**NT**–{**i**}=** T** *∪* {**i**}–{**i**} =** T**).
  - **i** ≡ ((**a**∧**b**) → **c**)≡((**b**∧**a**) → **c**):
    - (**a** ∈ **B**)⇒(**i** = **b** → **c**) ⇒ (then further analysis is like that in paragraph (a)),
    - (**b** ∈ **B**)⇒(**i** = **a** → **c**) ⇒ (then further analysis is like that in paragraph (a)),
    - (~**c** ∈ **B**)⇒(**i** = **a** → ~**b**)⇒ (then further analysis is like that in paragraph (a)),
    - (**a** ∈ **B**)∧(**b** ∈ **B**)∧(~**c** ∈ **B**)⇒** INCONS**,
    - (**a** ∉ **B**)∧(**b** ∉ **B**)∧(~**c** ∉ **B**)⇒ there is no reaction in this situation, so there is no impact for the final answer; we can hence treat that as (**NT**′=**NT**–{**i**}=** T** *∪*  {**i**}–{**i**}=**T**).
  - **i** ≡ ((**a**∧**b**)→(**c**∨**d**))≡((**b**∧ **a**)→(**d**∨**c**)):
    - (**a**  ∈**B**)⇒(**i**′ = (**b** → (**c**∨**d**))⇒ (then further analysis is like that in paragraph (b)),
    - (**b** ∈ **B**)⇒(**i**′ = (**a** → (**c**∨**d**))⇒ (then further analysis is like that in paragraph (b)),
    - (~**c** ∈ **B**)⇒(**i**′ = (**a**∧**b**) → **d**))⇒ (then further analysis is like that in paragraph (c)),
    - (~**d** ∈ **B**)⇒(**i**′ = (**a**∧**b**) → **c**))⇒ (then further analysis is like that in paragraph (c)),
    - (**a** ∈ **B**)∧(**b** ∈ **B**)∧(~**c** ∈ **B**)∧(~**d** ∈ **B**)⇒**INCONS**,
    - (**a** ∉ **B**)∧(~**b** ∉ **B**)∧(~**c** ∉ **B**)∧(~**d** ∉ **B**)⇒ there is no reaction in this situation so there is no impact for final answer; we can also treat this case as (**NT**′=**NT**–{**i**}=** =T** *∪*{**i**}–{**i**}=**T**).
  - Further possible implication schemes (up to 4 symbols) are (**a** → (**b**∧  **c**)), ((**a**∨**b**) → **c**), ((**a**∧**b**)→(**c**∧**d**)), ((**a**∨**b**)→(**c**∨**d**)), and ((**a**∧**b**)→(**c**∨**d**)) and they could be easily transformed to the schemes presented in paragraphs ((a)-(d)) above using the laws of logic presented in Section 2.1 and then analyzed accordingly. Implications including more than 4 symbols can be always decomposed to simpler formulas analyzed above due to the existence of normal forms of formulas described in Section 2 and transformation in Section 4.4.
3. Let us denote completeness of inference by C(**T**), where** T** is the set of other terms present in the test tube. In paragraph (1) above we demonstrated C(*⌀*), and in paragraph (2) we showed that, for any formula**w**, C(**T**)⇒ C(**T** *∪*{**w**}). According to the rule of mathematical induction we can take for granted also C(**T**) for an arbitrary** T** which ends this part of the proof.

For the proof of soundness, let us assume that the molecule representing positive answer emerged during the reactions in our test tube. One can observe the following.

1. When the molecule representing positive answer was produced in final test tube, it means that the molecules representing a basic fact and a question with mutually complementary sticky ends existed before. Only the molecules representing basic terms have restriction enzyme Bse*XI* in the position which can create the final molecule of positive answer.
2. If the molecule representing this basic term did not exist in** B** at the beginning, it had to be deducted by known implications whose correctness was proved in the first part of this proof. The terms received by inference have the same molecular representation (with enzyme Bse*XI* in the proper position) which was shown in Section 4.3.1.
3. There are no more possibilities of creating the molecule representing the positive answer because only the molecules representing facts (even received during some inferences) have enzyme Bse*XI* in the proper position. Observe that, although the longer artifact molecules described in Section 4.2 could eventually (although very unlikely) iterate to a length similar to that of a positive answer molecule, in this case they would be cut again by the enzyme Bse*XI*.

Due to the above given reasons, we deem our system as sound and complete. Using the laws of classical logic, it can run every possible inference and answer any question connected with it.

## 6. Computational Complexity

The experiment steps in laboratory can be briefly described as follows:

1. encoding every clause to DNA molecules,
2. mixing all the molecules in one test tube and leting all the possible ways of resolution be done automatically: looking for inconsistency; expanding the knowledge about facts and its value by* modus ponens* rule of deduction; and answering the questions,
3. filtering the result using (gel) electrophoresis: checking if the molecule signalizing inconsistency was created during the reaction; otherwise checking if some of the molecules connected with prepared questions were produced. The process ends here.

Steps 1 and 3 are constant time operations which means time complexity **O**(1). Step 2 needs more operations because it utilizes ligations and restriction reactions (regarded both as similar complexity). Looking for inconsistency in formula with* k*-variables needs maximally (*k* + 1) ligations and *k* restriction reactions; hence the time complexity of it is  **O**(*k*). Deductions and answering the questions are running in parallel at the same time and it needs maximally (*k* + 1) ligations and* k* restriction reactions too (in the worst case each of *k*-variables takes part in one deduction step; typically answering one question does not require knowledge about all variables). Hence the time complexity of step 2 is  **O**(*k*) and for the complete DNA algorithm it is also ***O***(*k*).

The space complexity is regarded as an asymptotic number of different DNA molecules (note that for proper reaction, each of them has to be present in many copies). Every fact, question, and simple implication needs exactly one molecule. For more complicated implications (using more than one literal in antecedent and/or more than one literal in consequent) it is better to prepare (*k*/2) molecules, because it lets every literal be represented by one sticky end, that is, in deduction rule (*a*∧*b* → *c*); there is no possibility of any resolution if we have knowledge only about fact *b*; but adding the second molecule representing (*b*∧*a* → *c*) solves the problem and resolution can be done even in this case. Theoretically in the most complicated situation there can be *k* rules of deductions, each represented by (*k*/2) molecules which gives the whole complexity  **O**(*k* ^2^). During the second step of algorithm, maximally every molecule can react with every molecule in subsequent inference steps (as it was already mentioned, each of them has to be present in many copies); the space complexity grows exponentially which means  **O**(2^*k*^).

## 7. Summary

In this paper we introduced a new DNA inference system based on the classical idea of splicing. It works with any formulae presented in special normal form which uses negation, conjunction, and implication. According to the laws of classical logic, every other formula can be transformed to such a form. The actual goal was to show that it is possible to run logical inference by DNA with two possible values of facts (*true*/*false*) which differ from the base concept of Shapiro's implementation of simple logic programs [19] which physically implements only one value.

Implementation of the most important laws of classical logic, which are necessary in connection with negation, was also presented so that the system is capable of any sequence of natural deduction steps. The presented model utilizes restriction enzyme Bse*XI* and a ligase enzyme so that its laboratory implementation is very similar to experiments already performed in [17, 19]. The system is autonomous; the only moment when human assistance is needed is preparing molecules before the reaction and reading the answer by gel electrophoresis after the reaction.

In conclusion, given that elementary reactions of splicing were laboratory-verified in [17, 19], we can assume that our new concept of compact inference system also works during the potential lab experiment.
